# Supplementary material for: The impact and return-on-investment of evidence-based practice in conservation and environmental management: A machine learning-assisted scoping review protocol
Source: PLoS One. 2025 Jun 25;20(6):e0326521. doi: 10.1371/journal.pone.0326521 (PMC12814508; doi:10.1371/journal.pone.0326521)
Supplement: S1 Appendix — (DOCX) [file pone.0326521.s001.docx]

# Appendix S1: PCC Framework (including alternate keywords used to construct search strings)

| PCC Framework Components | Main Concept or Subject | Alternate keywords |
| --- | --- | --- |
| Population | NA – see below. |  |
| Concept | Evidence-based practice | Evidence/Scientific evidence/ Research/Knowledge/Science AND use/based/guided/informed/based/led/driven/supported/ mobilisation/implementation/grounded/backed  Evidence-based conservation  Evidence-based environmental management  Evidence-based decision-making  Evidence implementation/synthesis/reviews  Knowledge transfer/translation/exchange  Use of evidence/research  Investment of research/evidence  Invest in research/evidence/knowledge/science  Evidence/science/research investment  Fund science/research/evidence  Fact-based/supported  Evidence integration |
|  | Evaluations and tests | test*  experiment*  trial*  pilot*  observ*  monitor*  survey*  sample*  implement*  control*  stud*  audit*  analy*  review*  compar*  investigat*  evaluat*  assess*  measur*  calculate* |
|  | Impact or effects | impact*  associat*  influence*  differ*  output*  gain*  decline*  increase*  decrease*  reduc*  enhance*  diminish*  advantage*  disadvantage*  productivity  trade-off  effect*  outcome*  result*  consequence*  benefit*  success*  failure*  harm*  perform*  achieve**  improve*  worse*  effectiveness  efficacy  change* |
|  | Return-On-Investment or Value-On-Investment | profit*  loss*  payoff*  return*  value*  cost*  ROI  VOI  return-on-investment*  value-on-investment*  value*  efficiency |
| Context | Conservation and environmental management  No geographical restriction | environmental manage*  natural resource manage*  wildlife manage*  landscape manage*  fisheries manage*  forestry manage*  ecological manage*  conservation manage*  ecosystem manage*  watershed manage*  coastal manage*  reserve manage*  park manage*  protected area manage*  agroecolog*  agricultural science*  environmental governance  environmental policy  conservation policy  water resource manage*  catchment manage*  river manage*  wetland manage*  grassland manage*  rangeland manage*  landscape ecology  species manage*  biodiversity manage*  environmental economics  ecological economics  natural hazard manage*  environmental health  environmental toxicology  environmental chemistry  conservation medicine  conservation psychology  conservation education  community-based conservation  community-based natural resource manage*  human-wildlife conflict  human wildlife conflict  environmental justice  wildlife crime  climate mitigation  climate adaptation  nature-based solution*  nature based solution*  biodiversity conservation  conservation biology  conservation science  conservation planning  wildlife conservation  habitat conservation  marine conservation  forest conservation  wilderness conservation  nature conservation  ecosystem conservation  ecological restoration  ecosystem restoration  landscape restoration  habitat restoration  applied ecology  restoration ecology  environmental protection  environmental stewardship  adaptive manage*  sustainable resource manage*  environmental assessment  environmental planning  land stewardship  natural resource stewardship  environmental studies  zoolog*  environmental science*  plant science*  soil science*  environmental engineering  environmental law  toxicolog*  species protection  habitat protection  biodiversity protection  ecosystem protection  environmental sustainability  ecological sustainability  integrated manage*  integrated environmental manage*  ecosystem-based manage*  landscape-scale conservation  ocean manage*  coastal zone manage*  lake manage*  pond manage*  stream manage*  estuarine manage*  resource conservation  natural area manage*  area manage*  wilderness preservation  species recovery  population manage*  animal manage*  fung* manage*  plant manage*  vegetation manage*  forest resource manage*  silviculture  agroforestry  conservation agriculture  regenerative agriculture  sustainable agriculture  soil conservation  soil manage*  forest manage*  ecosystem services manage*  conservation intervention*  conservation action*  conservation effort*  conservation initiative*  conservation measure*  conservation project*  biodiversity intervention*  restoration intervention*  preservation effort*  habitat intervention*  ecological intervention*  ecological action*  ecological initiative*  ecological measure*  ecosystem intervention*  ecosystem action*  ecological restoration*  ecological management action*  ecosystem management intervention*  environmental intervention*  environmental action*  environmental measure*  environmental management action*  environmental initiative*  sustainability intervention*  environmental restoration effort*  environmental improvement action*  environmental remediation*  climate intervention  climate action  climate measure*  climate engineering  climate mitigation effort*  climate remediation  climate-related initiative*  climate restoration effort*  climate adaptation intervention*  geoengineering intervention* |
