# Supplementary material for: The impact and return-on-investment of evidence-based practice in conservation and environmental management: A machine learning-assisted scoping review protocol
Source: PLoS One. 2025 Jun 25;20(6):e0326521. doi: 10.1371/journal.pone.0326521 (PMC12814508; doi:10.1371/journal.pone.0326521)
Supplement: S2 Appendix — (DOCX) [file pone.0326521.s002.docx]

# Appendix S2: Preliminary search terms for each bibliographic platform, database and search engine

| Web of Science Core Collection and CAB Abstracts:  TS=(  ("evidence-based" OR "evidence-informed" OR “evidence synthes*” OR “evidence review” OR “evidence-driven” OR “evidence-led” OR “evidence-guid*” OR “research-guid*” OR “research-led” OR “research-driven” OR “research-supported” OR “evidence-supported” OR “knowledge use” OR “knowledge transfer” OR “knowledge translation” OR “knowledge exchange” OR “knowledge implement*” OR “science-based” OR “science-informed” OR “knowledge mobili*” OR “evidence mobili*” OR “research mobili*” OR “science mobili*” OR "evidence use" OR "use of evidence" OR "evidence implementation" OR “research use” OR “use of research” OR “use of scien*” OR “research-informed” OR “research-based” OR “investment* of research” OR “investment* of evidence” OR “invest* in research” OR “invest* in evidence” OR “invest* in knowledge” OR “invest* in science” OR “evidence invest*” OR “science invest*” OR “research invest*” OR “fund* science” OR “fund* research” OR “fund* evidence” OR “evidence-backed” OR “fact-based” OR “fact-supported” OR “knowledge-supported” OR “knowledge-backed” OR “science-backed” OR “science-grounded” OR “research-supported” OR “research-backed” OR “evidence-grounded” OR “knowledge-grounded” OR “evidence integration”)  AND ("test*" OR "experiment*" OR “trial*” OR “pilot*” OR “observ*” OR “monitor*” OR “survey*” OR “sample*” OR “implement*” OR “control*” OR “stud*” OR “audit*” OR “analy*” OR "review*" OR "compar*" OR "investigat*” OR “evaluat*” OR "assess*" OR "measur*" or “calculat*”)  AND ("impact*" OR “associat*” OR “influence*” OR “differ*” OR “output*” OR “profit*” OR “loss*” OR “gain*” OR “decline*” OR “increase*” OR “decrease*” OR “reduc*” OR “enhance*” OR “diminish*” OR “advantage*” OR “disadvantage*” OR “payoff*” OR “return*” OR “value*” OR “productivity” OR “trade-off” OR “effect*” OR "outcome*" OR "result*" OR “consequence*” OR "benefit*" OR "success*" OR “failure*” OR “harm*” OR “cost*” OR "perform*" OR “achieve**” OR "improve*" OR “worse*” OR "ROI" OR "VOI" OR "return-on-investment*" OR "value-on-investment*" OR “value*” OR "efficiency" OR "effectiveness" OR "efficacy" OR "change*")  AND ("environmental manage*" OR "natural resource manage*" OR "wildlife manage*" OR "landscape manage*" OR "fisheries manage*" OR "forestry manage*" OR "ecological manage*" OR "conservation manage*" OR "ecosystem manage*" OR "watershed manage*" OR "coastal manage*" OR "reserve manage*" OR "park manage*" OR "protected area manage*" OR “agroecolog*” OR "agricultural science*" OR "environmental governance" OR "environmental policy" OR "conservation policy" OR "water resource manage*" OR "catchment manage*" OR "river manage*" OR "wetland manage*" OR "grassland manage*" OR "rangeland manage*" OR "landscape ecology" OR "species manage*" OR "biodiversity manage*" OR "environmental economics" OR "ecological economics" OR "natural hazard manage*" OR "environmental health" OR "environmental toxicology" OR "environmental chemistry" OR "conservation medicine" OR "conservation psychology" OR "conservation education" OR "community-based conservation" OR "community-based natural resource manage*" OR "human-wildlife conflict" OR "human wildlife conflict" OR "environmental justice" OR "wildlife crime" OR "climate mitigation" OR "climate adaptation" OR "nature-based solution*" OR "nature based solution*" OR "biodiversity conservation" OR "conservation biology" OR "conservation science" OR "conservation planning" OR "wildlife conservation" OR "habitat conservation" OR "marine conservation" OR "forest conservation" OR "wilderness conservation" OR "nature conservation" OR "ecosystem conservation" OR "ecological restoration" OR "ecosystem restoration" OR "landscape restoration" OR "habitat restoration" OR "applied ecology" OR "restoration ecology" OR "environmental protection" OR "environmental stewardship" OR "adaptive manage*" OR "sustainable resource manage*" OR "environmental assessment" OR "environmental planning" OR "land stewardship" OR "natural resource stewardship" OR "environmental studies" OR zoolog* OR "environmental science*" OR "plant science*" OR "soil science*" OR "environmental engineering" OR "environmental law" OR toxicolog* OR “species protection” OR “habitat protection” OR “biodiversity protection” OR “ecosystem protection” OR “environmental sustainability” OR “ecological sustainability” OR “integrated manage*” OR “integrated environmental manage*” OR “ecosystem-based manage*” OR “landscape-scale conservation” OR “ocean manage*” OR “coastal zone manage*” OR “lake manage*” OR “pond manage*” OR “stream manage*” OR “estuarine manage*” OR “resource conservation” OR “natural area manage*” OR “area manage*” OR “wilderness preservation” OR “species recovery” OR “population manage*” OR “animal manage*” OR “fung* manage*” OR “plant manage*” OR “vegetation manage*” OR “forest resource manage*” OR “silviculture” OR “agroforestry” OR “conservation agriculture” OR “regenerative agriculture” OR “sustainable agriculture” OR “soil conservation” OR “soil manage*” OR “forest manage*” OR “ecosystem services manage*” OR "conservation intervention*" OR "conservation action*" OR "conservation effort*" OR "conservation initiative*" OR "conservation measure*" OR "conservation project*" OR "biodiversity intervention*" OR "restoration intervention*" OR "preservation effort*" OR "habitat intervention*" OR "ecological intervention*" OR "ecological action*" OR "ecological initiative*" OR "ecological measure*" OR "ecosystem intervention*" OR "ecosystem action*" OR "ecological restoration*" OR "ecological management action*" OR "ecosystem management intervention*" OR "environmental intervention*" OR "environmental action*" OR "environmental measure*" OR "environmental management action*" OR "environmental initiative*" OR "sustainability intervention*" OR "environmental restoration effort*" OR "environmental improvement action*" OR "environmental remediation*" OR "climate intervention" OR "climate action" OR "climate measure*" OR "climate engineering" OR "climate mitigation effort*" OR "climate remediation" OR "climate-related initiative*" OR "climate restoration effort*" OR "climate adaptation intervention*" OR "geoengineering intervention*")  ) |
| --- |
| Scopus  TITLE-ABS-KEY (  ("evidence-based" OR "evidence-informed" OR “evidence synthes*” OR “evidence review” OR “evidence-driven” OR “evidence-led” OR “evidence-guid*” OR “research-guid*” OR “research-led” OR “research-driven” OR “research-supported” OR “evidence-supported” OR “knowledge use” OR “knowledge transfer” OR “knowledge translation” OR “knowledge exchange” OR “knowledge implement*” OR “science-based” OR “science-informed” OR “knowledge mobili*” OR “evidence mobili*” OR “research mobili*” OR “science mobili*” OR "evidence use" OR "use of evidence" OR "evidence implementation" OR “research use” OR “use of research” OR “use of scien*” OR “research-informed” OR “research-based” OR “investment* of research” OR “investment* of evidence” OR “invest* in research” OR “invest* in evidence” OR “invest* in knowledge” OR “invest* in science” OR “evidence invest*” OR “science invest*” OR “research invest*” OR “fund* science” OR “fund* research” OR “fund* evidence” OR “evidence-backed” OR “fact-based” OR “fact-supported” OR “knowledge-supported” OR “knowledge-backed” OR “science-backed” OR “science-grounded” OR “research-supported” OR “research-backed” OR “evidence-grounded” OR “knowledge-grounded” OR “evidence integration”)  AND ("test*" OR "experiment*" OR “trial*” OR “pilot*” OR “observ*” OR “monitor*” OR “survey*” OR “sample*” OR “implement*” OR “control*” OR “stud*” OR “audit*” OR “analy*” OR "review*" OR "compar*" OR "investigat*” OR “evaluat*” OR "assess*" OR "measur*" or “calculat*”)  AND ("impact*" OR “associat*” OR “influence*” OR “differ*” OR “output*” OR “profit*” OR “loss*” OR “gain*” OR “decline*” OR “increase*” OR “decrease*” OR “reduc*” OR “enhance*” OR “diminish*” OR “advantage*” OR “disadvantage*” OR “payoff*” OR “return*” OR “value*” OR “productivity” OR “trade-off” OR “effect*” OR "outcome*" OR "result*" OR “consequence*” OR "benefit*" OR "success*" OR “failure*” OR “harm*” OR “cost*” OR "perform*" OR “achieve**” OR "improve*" OR “worse*” OR "ROI" OR "VOI" OR "return-on-investment*" OR "value-on-investment*" OR “value*” OR "efficiency" OR "effectiveness" OR "efficacy" OR "change*")  AND ("environmental manage*" OR "natural resource manage*" OR "wildlife manage*" OR "landscape manage*" OR "fisheries manage*" OR "forestry manage*" OR "ecological manage*" OR "conservation manage*" OR "ecosystem manage*" OR "watershed manage*" OR "coastal manage*" OR "reserve manage*" OR "park manage*" OR "protected area manage*" OR “agroecolog*” OR "agricultural science*" OR "environmental governance" OR "environmental policy" OR "conservation policy" OR "water resource manage*" OR "catchment manage*" OR "river manage*" OR "wetland manage*" OR "grassland manage*" OR "rangeland manage*" OR "landscape ecology" OR "species manage*" OR "biodiversity manage*" OR "environmental economics" OR "ecological economics" OR "natural hazard manage*" OR "environmental health" OR "environmental toxicology" OR "environmental chemistry" OR "conservation medicine" OR "conservation psychology" OR "conservation education" OR "community-based conservation" OR "community-based natural resource manage*" OR "human-wildlife conflict" OR "human wildlife conflict" OR "environmental justice" OR "wildlife crime" OR "climate mitigation" OR "climate adaptation" OR "nature-based solution*" OR "nature based solution*" OR "biodiversity conservation" OR "conservation biology" OR "conservation science" OR "conservation planning" OR "wildlife conservation" OR "habitat conservation" OR "marine conservation" OR "forest conservation" OR "wilderness conservation" OR "nature conservation" OR "ecosystem conservation" OR "ecological restoration" OR "ecosystem restoration" OR "landscape restoration" OR "habitat restoration" OR "applied ecology" OR "restoration ecology" OR "environmental protection" OR "environmental stewardship" OR "adaptive manage*" OR "sustainable resource manage*" OR "environmental assessment" OR "environmental planning" OR "land stewardship" OR "natural resource stewardship" OR "environmental studies" OR zoolog* OR "environmental science*" OR "plant science*" OR "soil science*" OR "environmental engineering" OR "environmental law" OR toxicolog* OR “species protection” OR “habitat protection” OR “biodiversity protection” OR “ecosystem protection” OR “environmental sustainability” OR “ecological sustainability” OR “integrated manage*” OR “integrated environmental manage*” OR “ecosystem-based manage*” OR “landscape-scale conservation” OR “ocean manage*” OR “coastal zone manage*” OR “lake manage*” OR “pond manage*” OR “stream manage*” OR “estuarine manage*” OR “resource conservation” OR “natural area manage*” OR “area manage*” OR “wilderness preservation” OR “species recovery” OR “population manage*” OR “animal manage*” OR “fung* manage*” OR “plant manage*” OR “vegetation manage*” OR “forest resource manage*” OR “silviculture” OR “agroforestry” OR “conservation agriculture” OR “regenerative agriculture” OR “sustainable agriculture” OR “soil conservation” OR “soil manage*” OR “forest manage*” OR “ecosystem services manage*” OR "conservation intervention*" OR "conservation action*" OR "conservation effort*" OR "conservation initiative*" OR "conservation measure*" OR "conservation project*" OR "biodiversity intervention*" OR "restoration intervention*" OR "preservation effort*" OR "habitat intervention*" OR "ecological intervention*" OR "ecological action*" OR "ecological initiative*" OR "ecological measure*" OR "ecosystem intervention*" OR "ecosystem action*" OR "ecological restoration*" OR "ecological management action*" OR "ecosystem management intervention*" OR "environmental intervention*" OR "environmental action*" OR "environmental measure*" OR "environmental management action*" OR "environmental initiative*" OR "sustainability intervention*" OR "environmental restoration effort*" OR "environmental improvement action*" OR "environmental remediation*" OR "climate intervention" OR "climate action" OR "climate measure*" OR "climate engineering" OR "climate mitigation effort*" OR "climate remediation" OR "climate-related initiative*" OR "climate restoration effort*" OR "climate adaptation intervention*" OR "geoengineering intervention*")  ) |
| GreenFILE, ProQuest Dissertations & Theses, EBSCOhost Business Source Ultimate:  (("evidence-based" OR "evidence-informed" OR “evidence synthes*” OR “evidence review” OR “evidence-driven” OR “evidence-led” OR “evidence-guid*” OR “research-guid*” OR “research-led” OR “research-driven” OR “research-supported” OR “evidence-supported” OR “knowledge use” OR “knowledge transfer” OR “knowledge translation” OR “knowledge exchange” OR “knowledge implement*” OR “science-based” OR “science-informed” OR “knowledge mobili*” OR “evidence mobili*” OR “research mobili*” OR “science mobili*” OR "evidence use" OR "use of evidence" OR "evidence implementation" OR “research use” OR “use of research” OR “use of scien*” OR “research-informed” OR “research-based” OR “investment* of research” OR “investment* of evidence” OR “invest* in research” OR “invest* in evidence” OR “invest* in knowledge” OR “invest* in science” OR “evidence invest*” OR “science invest*” OR “research invest*” OR “fund* science” OR “fund* research” OR “fund* evidence” OR “evidence-backed” OR “fact-based” OR “fact-supported” OR “knowledge-supported” OR “knowledge-backed” OR “science-backed” OR “science-grounded” OR “research-supported” OR “research-backed” OR “evidence-grounded” OR “knowledge-grounded” OR “evidence integration”)  AND ("test*" OR "experiment*" OR “trial*” OR “pilot*” OR “observ*” OR “monitor*” OR “survey*” OR “sample*” OR “implement*” OR “control*” OR “stud*” OR “audit*” OR “analy*” OR "review*" OR "compar*" OR "investigat*” OR “evaluat*” OR "assess*" OR "measur*" or “calculat*”)  AND ("impact*" OR “associat*” OR “influence*” OR “differ*” OR “output*” OR “profit*” OR “loss*” OR “gain*” OR “decline*” OR “increase*” OR “decrease*” OR “reduc*” OR “enhance*” OR “diminish*” OR “advantage*” OR “disadvantage*” OR “payoff*” OR “return*” OR “value*” OR “productivity” OR “trade-off” OR “effect*” OR "outcome*" OR "result*" OR “consequence*” OR "benefit*" OR "success*" OR “failure*” OR “harm*” OR “cost*” OR "perform*" OR “achieve**” OR "improve*" OR “worse*” OR "ROI" OR "VOI" OR "return-on-investment*" OR "value-on-investment*" OR “value*” OR "efficiency" OR "effectiveness" OR "efficacy" OR "change*")  AND ("environmental manage*" OR "natural resource manage*" OR "wildlife manage*" OR "landscape manage*" OR "fisheries manage*" OR "forestry manage*" OR "ecological manage*" OR "conservation manage*" OR "ecosystem manage*" OR "watershed manage*" OR "coastal manage*" OR "reserve manage*" OR "park manage*" OR "protected area manage*" OR “agroecolog*” OR "agricultural science*" OR "environmental governance" OR "environmental policy" OR "conservation policy" OR "water resource manage*" OR "catchment manage*" OR "river manage*" OR "wetland manage*" OR "grassland manage*" OR "rangeland manage*" OR "landscape ecology" OR "species manage*" OR "biodiversity manage*" OR "environmental economics" OR "ecological economics" OR "natural hazard manage*" OR "environmental health" OR "environmental toxicology" OR "environmental chemistry" OR "conservation medicine" OR "conservation psychology" OR "conservation education" OR "community-based conservation" OR "community-based natural resource manage*" OR "human-wildlife conflict" OR "human wildlife conflict" OR "environmental justice" OR "wildlife crime" OR "climate mitigation" OR "climate adaptation" OR "nature-based solution*" OR "nature based solution*" OR "biodiversity conservation" OR "conservation biology" OR "conservation science" OR "conservation planning" OR "wildlife conservation" OR "habitat conservation" OR "marine conservation" OR "forest conservation" OR "wilderness conservation" OR "nature conservation" OR "ecosystem conservation" OR "ecological restoration" OR "ecosystem restoration" OR "landscape restoration" OR "habitat restoration" OR "applied ecology" OR "restoration ecology" OR "environmental protection" OR "environmental stewardship" OR "adaptive manage*" OR "sustainable resource manage*" OR "environmental assessment" OR "environmental planning" OR "land stewardship" OR "natural resource stewardship" OR "environmental studies" OR zoolog* OR "environmental science*" OR "plant science*" OR "soil science*" OR "environmental engineering" OR "environmental law" OR toxicolog* OR “species protection” OR “habitat protection” OR “biodiversity protection” OR “ecosystem protection” OR “environmental sustainability” OR “ecological sustainability” OR “integrated manage*” OR “integrated environmental manage*” OR “ecosystem-based manage*” OR “landscape-scale conservation” OR “ocean manage*” OR “coastal zone manage*” OR “lake manage*” OR “pond manage*” OR “stream manage*” OR “estuarine manage*” OR “resource conservation” OR “natural area manage*” OR “area manage*” OR “wilderness preservation” OR “species recovery” OR “population manage*” OR “animal manage*” OR “fung* manage*” OR “plant manage*” OR “vegetation manage*” OR “forest resource manage*” OR “silviculture” OR “agroforestry” OR “conservation agriculture” OR “regenerative agriculture” OR “sustainable agriculture” OR “soil conservation” OR “soil manage*” OR “forest manage*” OR “ecosystem services manage*” OR "conservation intervention*" OR "conservation action*" OR "conservation effort*" OR "conservation initiative*" OR "conservation measure*" OR "conservation project*" OR "biodiversity intervention*" OR "restoration intervention*" OR "preservation effort*" OR "habitat intervention*" OR "ecological intervention*" OR "ecological action*" OR "ecological initiative*" OR "ecological measure*" OR "ecosystem intervention*" OR "ecosystem action*" OR "ecological restoration*" OR "ecological management action*" OR "ecosystem management intervention*" OR "environmental intervention*" OR "environmental action*" OR "environmental measure*" OR "environmental management action*" OR "environmental initiative*" OR "sustainability intervention*" OR "environmental restoration effort*" OR "environmental improvement action*" OR "environmental remediation*" OR "climate intervention" OR "climate action" OR "climate measure*" OR "climate engineering" OR "climate mitigation effort*" OR "climate remediation" OR "climate-related initiative*" OR "climate restoration effort*" OR "climate adaptation intervention*" OR "geoengineering intervention*")  ) |
| Engineering Village expert search  ((("evidence-based" OR "evidence-informed" OR "evidence synthes*" OR "evidence review" OR "evidence-driven" OR "evidence-led" OR "evidence-guid*" OR "research-guid*" OR "research-led" OR "research-driven" OR "research-supported" OR "evidence-supported" OR "knowledge use" OR "knowledge transfer" OR "knowledge translation" OR "knowledge exchange" OR "knowledge implement*" OR "science-based" OR "science-informed" OR "knowledge mobili*" OR "evidence mobili*" OR "research mobili*" OR "science mobili*" OR "evidence use" OR "use of evidence" OR "evidence implementation" OR "research use" OR "use of research" OR “use of scien*” OR "research-informed" OR "research-based" OR "investment* of research" OR "investment* of evidence" OR "invest* in research" OR "invest* in evidence" OR "invest* in knowledge" OR "invest* in science" OR "evidence invest*" OR "science invest*" OR "research invest*" OR "fund* science" OR "fund* research" OR "fund* evidence" OR "evidence-backed" OR "fact-based" OR "fact-supported" OR "knowledge-supported" OR "knowledge-backed" OR "science-backed" OR "science-grounded" OR "research-supported" OR "research-backed" OR "evidence-grounded" OR "knowledge-grounded" OR "evidence integration") AND ("test*" OR "experiment*" OR "trial*" OR "pilot*" OR "observ*" OR "monitor*" OR "survey*" OR "sample*" OR "implement*" OR "control*" OR "stud*" OR "audit*" OR "analy*" OR "review*" OR "compar*" OR "investigat*" OR "evaluat*" OR "assess*" OR "measur*" or "calculat*") AND ("impact*" OR "associat*" OR "influence*" OR "differ*" OR "output*" OR "profit*" OR "loss*" OR "gain*" OR "decline*" OR "increase*" OR "decrease*" OR "reduc*" OR "enhance*" OR "diminish*" OR "advantage*" OR "disadvantage*" OR "payoff*" OR "return*" OR "value*" OR "productivity" OR "trade-off" OR "effect*" OR "outcome*" OR "result*" OR "consequence*" OR "benefit*" OR "success*" OR "failure*" OR "harm*" OR "cost*" OR "perform*" OR "achieve**" OR "improve*" OR "worse*" OR "ROI" OR "VOI" OR "return-on-investment*" OR "value-on-investment*" OR "value*" OR "efficiency" OR "effectiveness" OR "efficacy" OR "change*") AND ("environmental manage*" OR "natural resource manage*" OR "wildlife manage*" OR "landscape manage*" OR "fisheries manage*" OR "forestry manage*" OR "ecological manage*" OR "conservation manage*" OR "ecosystem manage*" OR "watershed manage*" OR "coastal manage*" OR "reserve manage*" OR "park manage*" OR "protected area manage*" OR "agroecolog*" OR "agricultural science*" OR "environmental governance" OR "environmental policy" OR "conservation policy" OR "water resource manage*" OR "catchment manage*" OR "river manage*" OR "wetland manage*" OR "grassland manage*" OR "rangeland manage*" OR "landscape ecology" OR "species manage*" OR "biodiversity manage*" OR "environmental economics" OR "ecological economics" OR "natural hazard manage*" OR "environmental health" OR "environmental toxicology" OR "environmental chemistry" OR "conservation medicine" OR "conservation psychology" OR "conservation education" OR "community-based conservation" OR "community-based natural resource manage*" OR "human-wildlife conflict" OR "human wildlife conflict" OR "environmental justice" OR "wildlife crime" OR "climate mitigation" OR "climate adaptation" OR "nature-based solution*" OR "nature based solution*" OR "biodiversity conservation" OR "conservation biology" OR "conservation science" OR "conservation planning" OR "wildlife conservation" OR "habitat conservation" OR "marine conservation" OR "forest conservation" OR "wilderness conservation" OR "nature conservation" OR "ecosystem conservation" OR "ecological restoration" OR "ecosystem restoration" OR "landscape restoration" OR "habitat restoration" OR "applied ecology" OR "restoration ecology" OR "environmental protection" OR "environmental stewardship" OR "adaptive manage*" OR "sustainable resource manage*" OR "environmental assessment" OR "environmental planning" OR "land stewardship" OR "natural resource stewardship" OR "environmental studies" OR zoolog* OR "environmental science*" OR "plant science*" OR "soil science*" OR "environmental engineering" OR "environmental law" OR toxicolog* OR "species protection" OR "habitat protection" OR "biodiversity protection" OR "ecosystem protection" OR "environmental sustainability" OR "ecological sustainability" OR "integrated manage*" OR "integrated environmental manage*" OR "ecosystem-based manage*" OR "landscape-scale conservation" OR "ocean manage*" OR "coastal zone manage*" OR "lake manage*" OR "pond manage*" OR "stream manage*" OR "estuarine manage*" OR "resource conservation" OR "natural area manage*" OR "area manage*" OR "wilderness preservation" OR "species recovery" OR "population manage*" OR "animal manage*" OR "fung* manage*" OR "plant manage*" OR "vegetation manage*" OR "forest resource manage*" OR "silviculture" OR "agroforestry" OR "conservation agriculture" OR "regenerative agriculture" OR "sustainable agriculture" OR "soil conservation" OR "soil manage*" OR "forest manage*" OR "ecosystem services manage*" OR "conservation intervention*" OR "conservation action*" OR "conservation effort*" OR "conservation initiative*" OR "conservation measure*" OR "conservation project*" OR "biodiversity intervention*" OR "restoration intervention*" OR "preservation effort*" OR "habitat intervention*" OR "ecological intervention*" OR "ecological action*" OR "ecological initiative*" OR "ecological measure*" OR "ecosystem intervention*" OR "ecosystem action*" OR "ecological restoration*" OR "ecological management action*" OR "ecosystem management intervention*" OR "environmental intervention*" OR "environmental action*" OR "environmental measure*" OR "environmental management action*" OR "environmental initiative*" OR "sustainability intervention*" OR "environmental restoration effort*" OR "environmental improvement action*" OR "environmental remediation*" OR "climate intervention" OR "climate action" OR "climate measure*" OR "climate engineering" OR "climate mitigation effort*" OR "climate remediation" OR "climate-related initiative*" OR "climate restoration effort*" OR "climate adaptation intervention*" OR "geoengineering intervention*")) WN KY) |
| Google:  ("evidence-based" OR "evidence-informed" OR "evidence use" OR "use of evidence" OR "use of scien*" OR “investment* of evidence” OR “invest* in evidence”)  ("environmental" OR "natur*" OR "ecolog*” OR "conservation*" OR "agri*" OR "climate" OR "adaptive manage*" OR “sustainability”)  ("impact*" OR “influence*” OR “effect*” OR “cost*” OR "ROI" OR "VOI" OR "return-on-investment*" OR "value-on-investment*" OR "change*")  ("test*" OR "experiment*" OR “trial*” OR “survey*” OR "compar*" OR “evaluat*” OR "assess*" OR "measur*" OR “calculat*”) |
| Google Scholar and Applied Ecology Resources:  ("evidence-based" OR "evidence-informed" OR "evidence use" OR "use of evidence" OR "use of scien*" OR “investment* of evidence” OR “invest* in evidence”)  AND ("environmental" OR "natur*" OR "wildlife" OR "ecolog*” OR "conservation*" OR "ecosystem*" OR "agri*" OR "biodiversity" OR "climate" OR "adaptive manage*" OR “sustainability”)  AND ("impact*" OR “influence*” OR “effect*” OR “cost*” OR "ROI" OR "VOI" OR "return-on-investment*" OR "value-on-investment*" OR "change*")  AND ("test*" OR "experiment*" OR “trial*” OR “survey*” OR "compar*" OR “evaluat*” OR "assess*" OR "measur*" OR “calculat*”) |
