# Supplementary material for: The impact and return-on-investment of evidence-based practice in conservation and environmental management: A machine learning-assisted scoping review protocol
Source: PLoS One. 2025 Jun 25;20(6):e0326521. doi: 10.1371/journal.pone.0326521 (PMC12814508; doi:10.1371/journal.pone.0326521)
Supplement: Attachment — Submitted filename: Response to Reviewers.docx [file pone.0326521.s005.docx]

PONE-D-25-03610

The impact and return-on-investment of evidence-based practice in conservation and environmental management: a machine learning-assisted scoping review protocol

***Overall response: We thank the editor and reviewers for their comments. We hope our responses and changes made to the manuscript are satisfactory. We give line numbers for our revisions in the tracked changes version of the manuscript (with changes not visible).***

***In addition to reviewer comments, we also highlight that we have updated the abstract screening methods to improve the structure and clarity of this section and make them even more methodologically rigorous – this includes approaches to balance the data, important for machine learning applications, and crucially including a more statistically justified stopping rule, instead of arbitrary stopping rules that are considered a pervasive issue in current review methods (Methods 2.3 Abstract screening and stopping criteria). We have also expanded the reviewer team by one member who has suggested improvements to the manuscript.***

***Editor’s comments:***

***Comment: The proposed scoping review will provide important insight into the value and impacts of evidence-based practice in environmental management and conservation. Although the proposed protocol appears sound, both reviewers made excellent suggestions that would strengthen the rationale and methodology.
Please address each of their specific questions and comments, ensuring that your revisions include:***

- ***A comparison EBP in medicine and conservation/environmental management, highlighting challenges/aspects unique to the latter context.***

***Response: We thank the editor for their comments and have now distinguished more between the differences of EBP in these different fields in the introduction (Introduction L71-96).***

***Comment:***

- ***Elaboration on what constitutes evidence in a conservation context and incorporate into your methodology for screening/study selection.***

***Response: We have now clarified more about what we mean by evidence in a conservation context in the introduction (Introduction L71-86) and incorporated this more into our review methods (Methods L383-384).***

***Comment:***

- ***Text related to the potential limitations of relying on literature to assess the extent to which evaluations of impacts and ROI of EBP in environmental/conservation management are conducted for human and environmental outcomes.***

***Response:  We have now elaborated on these limitations in our discussion (Discussion L412-418).***

***Reviewer 1 comments:***

***General comments***

***Comment:***  The overall aim of this study is to assess the impact and return on investment (ROI) of evidence-based practice (EBP) in conservation and environmental management through a scoping review. This is an important and timely study, as it seeks to fill a knowledge gap in evaluating the outcomes of EBP beyond healthcare, particularly in the environmental sciences. The concepts are well introduced in the Introduction section, with a clear rationale provided for the study. The Material and Methods section is detailed and adheres to established scoping review methodologies, including the use of PRISMA-ScR guidelines and AI-assisted screening tools (ASReview, FAST2). The Results are not yet available, as this is a protocol paper, but the proposed approach appears comprehensive. The discussion effectively highlights the potential significance of the study and its contributions to conservation decision-making.

***Response: We thank the reviewer for their constructive comments and the value they see in this proposed work.***

***Comment:***  However, there are a few areas that require further clarification and refinement. I, therefore, advise for a minor revision of the manuscript, including improvements in methodological transparency and addressing potential limitations. I provide specific comments below that I hope will be useful in improving the manuscript.

***Response: We hope the clarifications below help to address these areas for improvement.***

***Specific comments***

***Comment:***  The introduction clearly establishes the importance of EBP; however, it would benefit from a more structured comparison between the conservation and healthcare sectors in terms of evidence-based decision-making, as well as a more specific definition of EBP in the context of conservation. It is also related to the inclusion/exclusion criteria of your scoping review: How will you define that a study explicitly used some form of evidence? Won’t you miss concrete applications of conservation science evidence in policy and everyday management actions by using literature only? Don’t we also need interviews with conservation practitioners to have a sense of the use of EBP in conservation? Perhaps consider mentioning this as a perspective in your review?

***Response: These are valid points and this work forms part of a larger project that will use surveys and other approaches to obtain this data. This review however is important to understand what is currently out there in the literature to ensure we do not repeat effort. We have now clarified exactly what we mean by a study whereby evidence has been used (Methods L346-360) and to extract the type of evidence used to inform a change in practice (Methods L383-384). Our hypothesis is that little work on this has been published in the literature and therefore there is a pressing need for future research into this topic by doing exactly the sort of work you suggest in your comment.***

***Comment:***  The use of machine learning tools is innovative, but further details on their added value in systematic reviews would strengthen the methodological justification. What will they bring concretely, beyond saving some of your time?

***Response: A key thing that they will bring beyond productivity boost is the ability to potentially include relevant papers that a human reviewer might exclude. Humans are not perfect in conducting reviews and even when there is high agreement between reviewers there may still be systematic errors made. AI tools can help to potentially fill these gaps and retain high levels of performance.***

***Comment:***  The right panel of Figure 1 is confusing due to the use of general terms such as ‘LANGUAGE’ and ‘TYPE’, which are not specified.

***Response: Apologies the wrong Figure 1 was included in the submitted manuscript and we have now corrected this.***

***Comment:***  The authors acknowledge that scoping reviews do not assess the quality of included studies. However, given the study’s emphasis on ROI, will there be any considerations regarding the methodological rigor of economic assessments in conservation?

***Response: This is a good point. We will now include a description of how ROI was calculated to enable for future assessments of quality (Methods L385-386).***

***Comment:***  I thank the authors and editor for this interesting reading. As always, I sign this review and welcome the authors to engage with me on this topic. I’d be interested in learning more about your project and its future results.

***Response: Thank you for your review – we will be sure to follow up with you after the review process.***

***Reviewer 2:***

***General Comments***

***Comment:***  This protocol aims to understand the state of the evidence regarding impact and ROI of evidence-based practice in conservation and environmental management. The scoping review approach it describes is both clear and thorough. I think this work will make an important contribution to the field, which needs to better understand the evidence for, or lack thereof, impacts from evidence-based practice.

***Response: Thank you for your constructive review.***

***Comment:***  One other point I think that is worth mentioning in this protocol is how EBP is different in conservation vs. medicine. I think it should be pointed out that the nature of conservation interventions often do not lend well to randomized control trials. This does not mean we should give up or not try, but it does raise the issue of other approaches to evaluation to build the evidence base, such as counterfactuals; I think this notion should be mentioned somewhere in this paper and also likely should be a keyword for searches.

***Response: This is an important point and we have now tried to elaborate more on this as to how EBP in conservation differs to medicine (Introduction L71-96).***

***Specific Comments***

***Comment:***  Abstract: Line 17: There is an extra “appears to” here before “be”

***Response: Corrected (Abstract L17).***

***Comment:***  Lines 47-48: Consider providing references for the other “evidence-based” terms you list

***Response: We have now added references to other important evidence-based papers (Introduction L54).***

***Comment:***  Lines 51-52: What about the goal of more effective outcomes? This is perhaps the most important reason for EBP

***Response: Absolutely, we now add this in (Introduction L55-58).***

***Comment:***  Lines 71-72: In regards to not including “Indigenous knowledge in conservation and environmental management [24–26]” - this is an important shortcoming of some of the key systematic review guidance and I am pleased to see it highlighted here. The main stream guidance is typically lacking in providing guidance for including these other types of evidence. Some have pushed for a different terminology as a way to call attention to the need to include these other forms of evidence; could be worth pointing this out (e.g., Sterling et al. 2017 discusses using the broader more inclusive “evidence-informed” terminology instead of the more narrow “evidence-based” conclusion framing)

***Response: Good point – we now highlight this here (Introduction L86-89).***

***Comment:***  Lines 81-82: Seems here could point out how conservation interventions often vary in contexts, making it difficult to do randomized trials, etc. especially in comparison to say medicine

***Response: Yes good idea – we now point this out here (Introduction L89-96).***

***Comment:***  Lines 278-281: Should this also list change in cost as a human impact?

***Response: Yes good point, we have edited this (Methods L346-351).***
